# Allergic fungal rhinosinusitis presented as a unilateral nasal mass: A first case report from Thailand and literature review

**DOI:** 10.1016/j.amsu.2022.104855

**Published:** 2022-11-07

**Authors:** Jate Lumyongsatien, Pornsuk Cheunsuchon, Thiraphon Boonyaarunnate

**Affiliations:** a Department of Otorhinolaryngology - Head and Neck Surgery, Faculty of Medicine Siriraj Hospital, Mahidol University, Thailand; b Department of Pathology, Faculty of Medicine Siriraj Hospital, Mahidol University, Thailand

**Keywords:** Allergic fungal rhinosinusitis, Bent and Kuhn criteria, Southeast Asia, Case report

## Abstract

**Background:**

Allergic fungal rhinosinusitis (AFRS) is a relatively new inflammatory sinonasal disease. Prevalence of the disease is reported to be highly different across Asia.

**Case presentation:**

A 23-year-old Thai male came to our hospital with left-sided nasal obstruction. Endoscopic examination found a mass originated from the left sphenoethmoidal recess. Incisional biopsy result of the mass indicated an inflammatory process and high level of serum specific IgE to several aeroallergens was found. Based on the biopsy results and other investigations, the diagnosis of AFRS was made and the patient was treated successfully with endoscopic sinus surgery and postoperative systemic/topical steroids.

**Discussion:**

While AFRS is quite common in some regions, the disease is rarely encountered in Thailand and can be presented as a unilateral lesion, mimicking a tumor mass, which could lead to an incorrect diagnosis and inappropriate treatment.

**Conclusion:**

Even though AFRS is rarely reported in our country, it still can be found and might be recognized falsely as a neoplastic process. High level of awareness of the disease features could help to minimize inappropriate disease management.

## Highlights

• Prevalence of allergic fungal rhinosinusitis varies across Asia.
• Allergic fungal rhinosinusitis patients could present with sinonasal symptoms and signs mimicking neoplastic process.
• Awareness and early recognition of this condition could prevent unnecessary investigations and inappropriate treatments.

## Abbreviations

FRS =: Fungal rhinosinusitis
AFRS =: allergic fungal rhinosinusitis
CRSwNP =: chronic rhinosinusitis with nasal polyps

## 1 Introduction

Fungal rhinosinusitis (FRS) is an inflammatory disease of mucosa of the nasal cavity and paranasal sinuses and could be categorized into subgroups, based on interaction between hosts and fungal pathogens, as invasive and non-invasive FRS. The invasive group includes acute, chronic and granulomatous invasive FRS. Fungal ball, saprophytic fungal infestation and fungus-related eosinophilic FRS, including allergic fungal FRS (AFRS), are classified as non-invasive FRS [1]. AFRS is one type of chronic rhinosinusitis with nasal polyps (CRSwNP) that has distinctive features, marked by the presence of eosinophilic mucin and fungal hyphae in the paranasal sinuses with type I hypersensitivity to fungal antigens. The prevalence of AFRS might be subjected to geographic variation and could be as high as 27% in patients with CRSwNP [2,3]. In Asia, AFRS prevalence has been reported to be varied between regions and not commonly found in Southeast Asia [[3], [4], [5], [6], [7], [8], [9], [10], [11], [12]]. Specifically in Thailand, only 2 cases of AFRS have been reported so far, without details in clinical presentations, diagnostic findings and treatments [13]. In this case report, we present an AFRS patient with details in diagnosis and treatment at our institute. This work has been reported in line with the SCARE criteria [14].

## 2 Case presentation

**Fig. 1:**
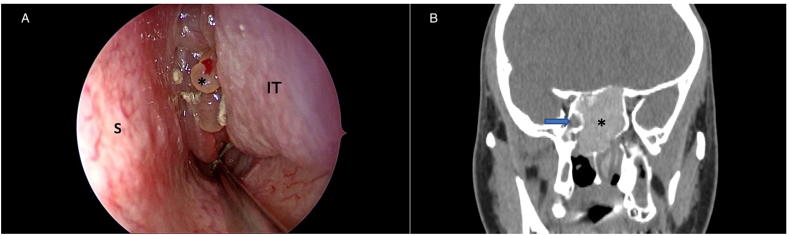
A) Polypoid mass *(asterisk)* in the left nasal cavity before surgery; S = nasal septum, IT = inferior turbinate; B) CT scan of the paranasal sinuses shows a hyperdense lesion *(asterisk)* in the left sphenoid sinus. The lesion expands the left sphenoid sinus cavity, making the size of the right sphenoid sinus cavity (*arrow*) extremely small.

**Fig. 2:**
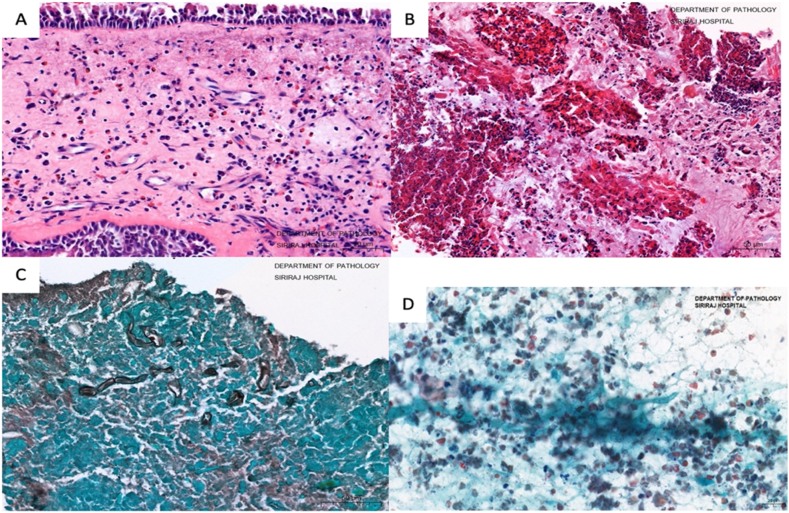
(A–B Hematoxylin and eosin stained, HE) The polyp stroma is inflamed and edematous. Inflammatory cells are a mixture of lymphocytes, plasma cells and eosinophils (A, ×20 and B, x40). Gomori methenamine silver (GMS) stain reveals few degenerated septate fungal hyphae (C, x60). The cellular smeared slide reveals rare tiny fungal hyphae-liked fragments on the background of debris and mucous material (D).

A 23-year-old Thai man presented with unilateral left nasal obstruction with increased left-sided nasal discharge for 1 month. A history of nasal allergy had been reported since childhood without asthmatic symptoms. No allergic symptoms in other family members were noted. General physical examination revealed nothing abnormal. On nasal endoscopy, there was a polypoid mass arising from the left sphenoethmoidal recess with some thick discharge on the surface of the mass (Fig. 1A). CT scan of the paranasal sinuses showed left posterior ethmoid and sphenoid sinus opacity with sinus wall expansion (Fig. 1B). The lesion seen in the CT scan was inhomogeneous and hyperdense without further enhancement after contrast media injection. The complete blood count was normal with 7.0% eosinophil count. Serum total IgE level was 3540 IU/ml (normal adult level <100 IU/ml). Serum specific IgE was positive for *Aspergillus*, mixed mold, house dust mite and cockroach antigens. Due to the scarcity of reported AFRS in our country and the purely unilateral picture of the lesion, a neoplastic process was initially suspected and biopsy of the polypoid mass was done to make a definite diagnosis. The biopsy result was inflammatory polyp. After obtaining the histological study result, the diagnosis of AFRS was made and the patient was offered sinus surgery as a definite surgical treatment. Endoscopic sinus surgery was performed by the attending ENT surgeon (JL) in our tertiary hospital and polypoid change of the sinus mucosa with thick greenish-brown discharge were found intraoperatively, mainly in the left sphenoid sinus. As expected, no tumor mass was detected. The abnormal discharge was removed totally, the polyps were taken out as much as possible and the sinus openings were opened widely to facilitate postoperative topical therapy. The postoperative period was uneventful. The final histological result of the polyps was allergic polyp without demonstrable fungal tissue invasion. The mucinous discharge was eosinophilic in nature and was positive for fungal fragments which were branching septate hyphae on Gomori methenamine silver stain (Fig. 2A–D).

After the operation, the patient was prescribed a tapering course of oral prednisolone (starting from 0.5 mg/kg/day), intranasal fluticasone furoate spray (27.5 μg/spray) 2 puffs to each nostril twice daily and budesonide nasal irrigation once daily (budesonide 1mg mixed with normal saline solution 200ml). The patient started to feel better a few days postoperatively with only minor epistaxis and nasal obstruction. A significant improvement was reported by the patient after 2 weeks of systemic steroid administration. The decision to decrease the dose of oral prednisolone and other topical treatments was based on the patient's symptoms, endoscopic findings and serum total IgE level. Finally, oral prednisolone and budesonide nasal irrigation could be stopped at 3 months after surgery, with the serum total IgE level decreased from 3540 IU/ml to 1460 IU/ml. During and after cessation of the systemic steroid treatment, no obvious side effects of the medicine were observed. Topical intranasal corticosteroid spray was given to use for a longer period to control his allergy symptoms. On the last visit, 50 months after surgery, the patient had only clear nasal discharge occasionally and nasal endoscopic examination revealed normal sinus mucosa without polyps or thick discharge.

## 3 Discussion

Firstly described in 1976, AFRS is now considered as one phenotype of CRSwNP, characterized by eosinophil-rich mucin, presence of non-invasive fungal hyphae in the paranasal sinuses and type I hypersensitivity to fungi. Although some controversy still exists regarding diagnosis, the original Bent and Kuhn 5 major criteria are the most logical and widely used as a standard diagnostic tool; 1) nasal polyposis, 2) presence of fungi on fungal staining, 3) eosinophilic mucin without fungal invasion into the sinus tissue, 4) type I hypersensitivity to fungi and 5) CT showed areas of high attenuation in the sinuses [15]. Several mechanisms have been recognized to be potentially responsible for the development of this condition, including group 2 innate lymphoid cell immune responses (ILC2s), dysregulation of regulatory T-cell and CD-8^+^ T-cell function, increased activity of Th17 cells [16]. Different geography and climate might also have significant roles in AFRS development, along with genetics of the population. For example, the prevalence of AFRS in the United States were reported to differ from region to region, ranged from 0% to 8.2% of CRS patients who required surgery [2]. HLA-DQB1*0301 and HLA-DQB1*0302 were found more frequently in AFRS patients compared to patients with hypertrophic sinus disease and healthy people [17]. These differences could be among several reasons that make the prevalence of AFRS diverse so highly in our vast Asian continent (Table 1).

**Table 1:** Prevalence of AFRS in Asian countries; all studies used the original Bent and Kuhn criteria for diagnosis.

Study	Country of origin	AFRS/surgical CRS	% Prevalence
Bakshsaee et al. [4]	Iran	12/141	8.51%
Aeumjaturapat et al. [13]	Thailand	2/214	0.93%
Alshaikh et al. [11]	Singapore	5/591	0.85%
Challa et al. [18]	India	15/63	23.8%
Dhanani et al. [6]	Pakistan	27/114	23.68%
Telmesani [9]	Saudi Arabia	11/91	12.09%
Demir et al. [5]	Turkey	0/87	0%
Goh et al. [3]	Malaysia	8/30	26.67%
Makihara et al. [12]	Japan	6/429	1.4%

Clinically, AFRS patients mostly present with the same picture as in CRSwNP, except AFRS cases tend to have more severe symptoms and are more difficult to treat. Additionally, AFRS is more likely to occur in the adolescents or young adults and usually has bilateral, but asymmetrical, sinus lesions [15]. Detailed examination of sinonasal discharge will find eosinophils and positive fungal hyphae, which are among the diagnostic criteria of the disease. Of note, positive fungal culture could not be used as an evidence of a fungal-related condition as fungal contamination could be found in nearly 100% in both normal and CRS subjects [19]. Despite the fact that local IgE might have a role, usually patients with AFRS have high total serum IgE level and increased specific serum IgE level to multiple fungal antigens [16].

Treatment of AFRS consists of sinus surgery and medical therapy. The aims of surgery are removing fungal antigens and providing openings into the sinus cavities to facilitate topical treatments postoperatively. For the medical treatment, oral and topical intranasal steroid sprays are widely agreed to be the effective medicine with cautions of the side effects, both in short and long term, of the oral steroids [20]. The duration and dose of oral steroid administration is subjected to severity of the disease and must be judged on a case-by-case basis. High-volume, low-pressure steroid irrigation has been demonstrated to be safe and could be used in AFRS also, even though this kind of topical steroid administration is still off-label [20].

In this report, we describe an AFRS patient who presented with a rare unilateral lesion and responded well to surgery and postoperative conventional treatments. However, it is still difficult to predict that other AFRS patients in our country will be treated successfully with the same treatment protocol as the data about the disease is still very limited.

## 4 Conclusion

Although AFRS is obviously rare in Thailand, we provide details that this illness does exist in our country and should be included in the differential diagnosis when appropriate. As symptoms and signs of AFRS are comparable to CRSwNP, early recognition of this condition could minimize both direct and indirect burdens relating to the disease. Further studies, such as genetics or environmental research, could be conducted to gain more explanations about the factors influencing disease development.

## Consent

Written informed consent was obtained from the patient.

## Author note

We have no known conflict of interest to disclose.

## Ethical approval

The study was conducted in accordance with the principles laid down in the Declaration of Helsinki. Written informed consent was obtained from the patient for publication of this case report and accompanying images.

## Sources of funding

The authors declare that no funding was obtained for this study.

## Author contribution

Jate Lumyongsatien: study concept, data collection, data interpretation, writing and correction of the manuscript; Pornsuk Cheunsuchon: data collection and interpretation; Thiraphon Boonyaarunnate: data collection and interpretation.

## Trail registry number

1. Name of the registry: Thai Clinical Trials Registry.

2. Unique Identifying number or registration ID: TCTR20220809004.

2. Hyperlink to your specific registration (must be publicly accessible and will be checked): https://www.thaiclinicaltrials.org/show/TCTR20220809004.

## Guarantor

Jate Lumyongsatien.

## Provenance and peer review

Not commissioned, externally peer-reviewed.

## Declaration of competing interest

The authors declare that they do not have any conflicts of interest.
